# Disparate Effects of Invasive Group A *Streptococcus* on Native Americans

**DOI:** 10.3201/eid2609.181169

**Published:** 2020-09

**Authors:** Ryan M. Close, James B. McAuley

**Affiliations:** University of Pennsylvania, Philadelphia, Pennsylvania, USA (R.M. Close);; Indian Health Service Hospital, Whiteriver, Arizona, USA (R.M. Close, J.B. McAuley);; Rush University, Chicago, Illinois, USA (J.B. McAuley)

**Keywords:** Streptococcus pyogenes, American Indian, Alaska Native, Indigenous, bacteria, group A Streptococcus, Australia, United States, Canada, New Zealand, streptococci, Native Americans, GAS, iGAS

## Abstract

Active surveillance of invasive group A *Streptococcus* (iGAS) disease indicates that its incidence in the US general population is low, but limited studies show rates for American Indians and Alaska Natives (AI/AN) are severalfold higher. Major disparities in rates of iGAS exist between Indigenous and non-Indigenous populations of Australia, New Zealand, and Canada, but much less is understood about iGAS among AI/AN in the United States. Although complex host–pathogen interactions influence the rates of iGAS, including strain variation and virulence, the number and type of concurrent conditions, and socioeconomic status, the relative contribution of each remains unclear. We highlight the poor correlation between the substantial effect of iGAS among Indigenous persons in industrialized countries and the current understanding of factors that influence iGAS disease in these populations. Prospective, large-scale, population-based studies of iGAS are needed that include AI/AN as a necessary first step to understanding the effects of iGAS.

*Streptococcus pyogenes* (group A *Streptococcus* [GAS]) causes a range of pyogenic, toxigenic, and immunologic diseases with varying degrees of severity, from pharyngitis and impetigo to necrotizing fasciitis and streptococcal toxic shock syndrome (*1*). Improved sanitation and increased availability of antimicrobial drugs have led to a decline in GAS incidence and virulence (*2*), but concern about invasive GAS (iGAS) has increased in recent decades (*3*).

Each year, ≈500,000 deaths occur worldwide from GAS; 160,000 of these are from invasive infections, of which 97% occur in resource-limited countries (*4*). Some of the highest rates of iGAS occur in Indigenous populations within industrialized countries (*5*), most likely as a consequence of health disparities between general populations and marginalized peoples, in addition to a poorly understood host–pathogen relationship. In the United States, American Indians and Alaska Natives (AI/AN) suffer from high rates of iGAS, but epidemiologic and clinical data on GAS among these Indigenous populations is more sparse than it is in other industrialized nations (*6*). We highlight discordance between the substantial effect of iGAS on AI/AN and understanding of factors that influence iGAS disease in these vulnerable US populations and in other Indigenous persons.

## iGAS in the United States

During the 1980s, the first reports of streptococcal toxic shock–like syndrome resulted in a resurgence in concern about GAS disease. A population-based study of iGAS in the United States found a stable annual incidence rate of 4.3 cases/100,000 population during 1985–1990 (*7*). Despite this stable rate, GAS disease increased, and multiorgan dysfunction and hypotension signaled an increase in GAS severity.

This study prompted the Centers for Disease Control and Prevention (CDC) to add iGAS to the Active Bacterial Core surveillance (ABCs) program. The first survey, which assessed iGAS in the United States during 1995–1999, found an annual incidence of 2.2–4.8 iGAS cases/100,000 population (*3*). Rates were stable in 2 follow-up epidemiologic studies that showed iGAS incidence of 3.5 cases/100,000 population for 2000–2004 and 3.8 cases/100,000 population for 2005–2012 (*1*,*8*).

The CDC reports suggested stability with unchanging rates and disease distribution. Each report concluded that iGAS incidence had been stable not only intrastudy but also across decades, starting with the work of Hoge et al. (*7*). The researchers referenced higher rates of iGAS among Indigenous populations of other countries, and ABCs data indicated that AI/AN make up <2% of the country but contributed 4% of iGAS cases during 2000–2012 (*1*,*8*). This finding provides at least inferential evidence of higher rates of iGAS among AI/AN that warrants further investigation.

Incidence varies by region. For example, Utah reported an iGAS incidence significantly higher than country averages for 2002–2010: 6.3 cases/100,000 Utah residents. The study noted an increased relative risk among Native Hawaiians/Pacific Islanders (3.3% vs. 1.2%), but this observation was not explored further (*9*). More recent data from the ABCs program indicated an increase in iGAS incidence in the general population; rates rose in 2014 to 4.8 cases/100,000 population and in 2016 to 5.8 cases/100,000 population (*10*,*11*). Although this recent increase is notable, it is marginal, and overall rates remain substantially lower than those reported among AI/AN.

These studies highlight the stable pattern of iGAS in the general US population. However, they fail to highlight the limited but important evidence that suggests higher iGAS rates among AI/AN.

## iGAS among AI/AN

Hoge et al. reported an overall iGAS incidence of 4.3 cases/100,000 population but found an age-adjusted incidence of 46.0 cases/100,000 population for AI (*7*). Heightened concern for iGAS among AI led Benjamin et al. to review all cultures positive for GAS from sterile sites at a hospital serving Zuni Indians during the same period (1982–1991) (*12*). The annualized incidence rate for iGAS of 13.3 cases/100,000 population (95% CI 8.3–33.2) for Zuni Indians contrasted with a rate of 1.7 cases/100,000 population in a neighboring county of predominantly Hispanic and non-Hispanic White residents. The authors noted that during a time when the medical community was focused on suspected rising rates in the general population that remained in single digits, the rate of iGAS among Zuni AI patients was, and had been, substantially higher.

The findings are consistent with more recent studies and case series that described iGAS among AI/AN. Rudolph et al. evaluated iGAS in Alaska during 2001–2013, and although only 20% of the state’s population was AN, this group represented 46% of the state’s 516 iGAS cases. The rates of iGAS were 13.7 for AN and 3.9 for non-AN persons per 100,000 population (*6*). The Arizona Department of Health Services reported iGAS incidence rates for AI/AN that were 2–5 times higher than those for the general population and an increase in rates in recent years (*13*). However, this information has not reached the broader public health or medical literature that mostly comprises smaller, local reports of iGAS clusters involving AI communities (*14*,*15*).

## A Disease of Displaced Indigenous Populations

Other high-resource countries with more recent iGAS population-based studies among Indigenous populations can provide insight for an explanatory model for iGAS among Indigenous persons in the United States. Although evidence is limited on the true incidence of iGAS globally, Indigenous populations of industrialized nations have the highest recorded rates of both epidemic and endemic iGAS disease (*4*).

### Canada

After an iGAS outbreak swept through Canada, researchers conducted an epidemiologic review of iGAS cases in northwestern Ontario for 2001–2013 at the health center, metropolitan, and provincial levels (*16*). First Nations Canadians accounted for 41% of iGAS cases despite making up only 8% of the population. The rate of iGAS at the health center where most of this population obtains care ranged from 9.8 to 18.1 cases/100,000 population, a rate 2–7 times higher than the largely nonnative metropolitan and provincial populations. In a more focused follow-up study of 22,000 First Nations Canadians among 26 communities in the same region, Bocking et al. reported an iGAS annualized rate of 56.0 cases/100,000 population for 2009–2014, >10 times higher the rate for the general population during the same period (*17*).

### Australia

The rates of GAS and poststreptococcal sequelae are substantially higher for Aboriginal Australians than for the general population of Australia, for which GAS disease has declined (*18*). In a retrospective 6-year study of all cases of GAS bacteremia in the Northern Territory, Carapetis et al. noted that >50% of cases occurred in Aboriginal Australians, who make up only one quarter of the population (*19*). Two separate sequential studies in neighboring Queensland spanning 1996–2009 supported these findings; rates of iGAS for Aboriginal Australians were 3–8 times higher than for non-Indigenous Australians (Appendix).

### New Zealand

In nearby New Zealand, Safar et al. conducted a population-based study of iGAS to anticipate coverage of potential GAS vaccines in development (*20*). In the diverse city of Auckland (population ≈1.3 million), 11% were Indigenous Māori. Yet, this group accounted for 31% of all iGAS infections over a 2-year period, and the incidence rate for Māori was 5 times higher than for New Zealand residents of European and Asian descent. The disparity was more dramatic at the ends of the age spectrum for Indigenous Māori. iGAS incidence rates per 100,000 population were 40.9 for children <1 year of age and 146.8 for persons >65 years of age.

The disparity in iGAS between Indigenous and non-Indigenous populations spans geography and cultures (Table). However, the extent and reasons for that disparity remain poorly understood.

## Influencing Factors in iGAS

The relationship between GAS and Indigenous populations most likely involves complex interactions between pathogens and humans. Numerous studies have contributed to a long list of influencers of GAS incidence but a lack of rigorous analyses has yielded few prevention strategies (*21*). The high rates of iGAS among Indigenous persons may represent unique circumstances that combine pathogen characteristics, population dynamics, and individual risk factors that all contribute to varying degrees.

A high incidence of noninvasive GAS disease and postinfectious sequelae is well described in the Indigenous populations of industrialized countries and coincides with elevated rates of iGAS (*5*). Among Indigenous Australian children, the prevalence of pyoderma was reported as high as 90% (*18*), and incidence rates were 4–10 times higher than in other resource-limited settings (*22*). Rates of acute rheumatic fever for Indigenous Australians are as high as 194 cases/100,000 population and for Māori in New Zealand, 78 cases/100,000 population (*5*). These rates are down from estimated rates of 374–508 cases/100,000 population during the 1980s, but remain higher than rates for local non-Indigenous groups (1.1–7.2/100,000) (*5*). Finally, Indigenous Australians have the highest reported rates of acute poststreptococcal glomerulonephritis worldwide (239 cases/100,000 population) (*23*).

Less is known about noninvasive GAS and post-GAS sequelae among AI/AN in recent decades. Studies among AI children in Minnesota during the 1960s and 1970s showed high rates of poststreptococcal glomerulonephritis in addition to a >80% prevalence of pyoderma (*24*,*25*). Despite national declines in rheumatic heart disease, limited reports reveal rates of rheumatic heart disease in AI nearly 5 times national estimates (4.6 vs. <1.0 cases/1,000 population) (*26*) along with higher rates of rheumatic heart disease–related death (*27*). These observations require a broader appreciation of GAS among Indigenous persons, including AI/AN.

### Socioeconomic Status

Socioeconomic status (SES) is a commonly asserted but poorly studied explanation for the high rates of iGAS among Indigenous persons. Few studies have directly measured SES when comparing iGAS rates between Indigenous and non-Indigenous populations, and the evidence is conflicting. In 1 study from Australia, half of iGAS cases occurred in the lowest socioeconomic quintile, in which Indigenous persons were overrepresented (*20*). Yet, a study in Fiji that specifically looked for a relationship with SES found that Indigenous Fijians had higher rates of iGAS despite a higher SES than did Indo-Fijians (*28*). Although this study was limited by small sample sizes, it is an example of the difficulty in understanding how SES indicators relate to iGAS incidence. Yet, of all the potential influencers, SES seems to be the most obvious commonality between displaced Indigenous persons of different industrialized nations.

According to US national health statistics, 23.9% of AI/AN lived below the poverty level during 2014–2016 (*29*), whereas 12.7% of all persons and 11.0% of White persons lived below the poverty level (*29*). According to Akee et al., AI/AN experience substantial income inequality and immobility. Along with Blacks and Hispanic persons, AI/AN are consistently at the lower end of income distribution in the United States and are the least likely to move percentiles in their lifetime (*30*). Of the top 10% of US income earners, 84% were White, whereas only 0.3% were AI/AN (*30*).

For numerous health indicators, AI/AN are worse off as well and have some of the highest age-adjusted death rates secondary to chronic liver disease and cirrhosis, diabetes, unintentional injuries, septicemia, and alcohol-related deaths (*29*). Although the overall age-adjusted death rate for AI/AN is lower than that for all races (591 vs. 729/100,000 population), the years of potential life lost before age 75 years is 10% higher for AI/AN than for White persons (i.e., AI/AN lose 645 more years of life per 100,000 persons before age 75 years) (*29*). Infant mortality is a “fundamental indicator of … community health status, and the availability and use of appropriate health care,” and from 2005 to 2015, infant mortality rates decreased >10% for every racial group except AI/AN, for whom infant mortality rates did not improve (*29*).

For some conditions, however, AI/AN have lower death rates than the US general population and for White Americans. These conditions include heart disease, cerebrovascular disease, malignancies, and chronic lower respiratory disease (*29*). Misclassification and incomplete data for racial groups other than White and Black explains some of the reason for these lower death rates.

Although AI/AN experience socioeconomic and health disparities, linking the 2 causally is complicated. The relationship between SES overall, specific SES indicators (e.g., income or education), and health outcomes varies by race (*31*). Furthermore, Black Americans have near equivalent rates of poverty (22.0% in 2016) to AI/AN and have some of the largest health disparities of any racial group (*29*). The all-cause age-adjusted death rate is highest for Black persons (857/100,000 population), and Black persons have higher death rates for heart disease, cerebrovascular disease, malignancies, diabetes, and homicides than persons of all other races (*29*). Although Black Americans have worse health indicators than AI/AN for many conditions, the rates of iGAS among Blacks are more similar to those among Whites than for AI/AN. In the most recent comprehensive report by CDC, the rate of iGAS among Blacks was 4.7 cases/100,000 population and increased to 6.2 cases/100,000 population in the 2016 update (*1*,*11*).

For these reasons, we caution against attributing substantial disparities in iGAS among AI/AN compared with the general population to SES alone. Although SES undoubtedly plays a major role, more information is needed on which SES indicators most strongly predict iGAS in AI/AN and the general population. Such information will best guide public health interventions beyond oversimplified suggestions to improve hygiene and ameliorate poverty.

Perhaps the reason SES fails to accurately capture which, and how, certain SES indicators most affect Indigenous persons is that the colonization, displacement, assimilation practices, and subsequent intergenerational historical trauma experienced by Indigenous populations fundamentally differentiate them from other impoverished groups. The health effects of centuries of denying and denigrating Indigenous culture have only recently been appreciated, but much needs to be done to promote healing (*32*). The displacement and discrimination experienced by AI/AN is difficult to measure and correlate with health outcomes. Furthermore, the experiences of Indigenous persons have shaped migration, their interactions with non-Indigenous communities, and relationships with institutions such as the education and healthcare systems. These factors have led to social, economic, and political disparities not captured in typical epidemiologic studies and may contribute to the confusion in how SES is linked to health disparities among AI/AN. Recognizing these historical differences for AI/AN is critical for better understanding health disparities, such as iGAS, and might be the most important and least understood aspect of disparities in Indigenous health.

### Microbiological Factors

Although strain novelty and virulence play important roles in iGAS outbreaks, their differential contribution remains unclear. Some studies have postulated that strain prevalence and novelty, rather than innate virulence potential, was the predominant reason a specific strain would emerge as a cause of iGAS (*33*). That is, upsurges in both iGAS and noninvasive GAS disease could occur when a predominant strain is supplanted with a new *emm*-type to which the population lacks sufficient immunity (*21*,*34*). Outbreaks can occur within specific risk groups that may implicate interaction between strain-type and host irrespective of virulence (e.g., the spread of severe GAS infections among intravenous drug users in the United Kingdom) (*35*).

Yet, hypervirulent strains also have contributed to outbreaks of iGAS (*36*,*37*). A well-described outbreak of iGAS in Canada resulted from a single, recently emerged, hypervirulent strain of *emm59*, which then migrated to the United States; genetically diversified; and led to *emm59* outbreaks in Montana, Wyoming, and Arizona (*15*,*38*,*39*). During the outbreak in Canada, Tyrrel et al. noted that a relatively modest proportion of iGAS cases (5%) in Ontario was attributable to *emm59*. Yet, they observed that 56 (83%) of Ontario’s 68 *emm59* cases occurred in an area with a high proportion of First Nations Canadians (*37*). This observation supported by the findings of Athey et al. that reported a disproportionate number of iGAS cases among Indigenous Canadians in Ontario, particularly in the Thunder Bay region, where *emm59* caused 44% of iGAS in 2008 (*16*). An *emm59* outbreak in a northern Arizona hospital cited Native American race as a primary risk factor for infection. Eighteen (62%) of 29 iGAS cases were identified as *emm59*, 15 (83%) of which occurred in AI (*15*). A phylogenetic analysis of 67 *emm59* strains known to cause outbreaks of iGAS in the United States revealed that AIs had the highest proportion of invasive *emm59* infections (*40*). However, without more detailed community epidemiologic data, the relative contribution of novelty versus virulence was difficult to discern. Genetic studies of invasive streptococcal outbreaks in the United States suggest that a progenitor pathogen makes its way into a population (e.g., AI/AN) and then spreads quickly from person to person if the dynamics are favorable (*15*,*16*,*39*,*40*). Novelty and virulence most likely work together to intensify iGAS epidemics in Indigenous communities. Where a new pathogen might lead to an increase in iGAS overall because of population susceptibility, a particularly virulent new pathogen leads to dramatically increased rates of disease. It is entirely plausible that population-specific dynamics and pathogen virulence work together in amplifying outbreaks.

### Host Factors: Concurrent Conditions and Immunologic Vulnerability

Evidence suggests a strong relationship between iGAS and the prevalence of concurrent conditions (*19*). Diabetes, skin disease, chronic kidney disease, heart disease, and alcoholism are consistently associated with iGAS (*1*,*17*,*20*,*28*). Although some studies found Indigenous persons with iGAS were more likely than non-Indigenous persons to have diabetes, chronic kidney disease, or both (*7*,*19*), evidence demonstrating no association in the number or type of concurrent conditions was equally limited (*20*,*28*). This observation reflects the limitations of the research and the poor understanding of how concurrent conditions contribute to iGAS. Indigenous populations clearly have more such conditions and acquire them at a younger age (*41*,*42*). The quantity of concurrent conditions, in addition to the specific conditions (e.g., diabetes), might explain why Indigenous persons, including AI/AN, are overrepresented among iGAS cases and is supported by the higher rates of all-cause infectious disease–related death, including from iGAS, in AI/AN (*43*). However, this approach would be an oversimplification that neither accounts for the role of pathogen virulence and host factors nor provides insight into how such conditions specifically contribute to iGAS. Furthermore, attributing markedly elevated rates of iGAS to overall vulnerability cannot explain the variability in findings on the effects of concurrent conditions among surveys. Concurrent conditions appear to play an integral but incomplete role in the incidence of iGAS among Indigenous populations. Prospective studies to interpret the effects of specific conditions on the incidence of iGAS are needed to better identify persons at highest risk within vulnerable populations.

The potential contribution of host genetics is the least understood of variables. Evidence suggests genetic conservation among AI/AN (*44*), and a genetic basis for several conditions prevalent among AI/AN has been explored or established, including asthma (*45*), diabetes (*46*), and rheumatoid arthritis (*47*). Evolutionary bottlenecks and founder effects have led to an overall decrease in allele diversity, and tribal structure of reservation life has led to semi-independent gene pools (*44*,*48*). Such gene pools provide a potentially more homogenous widespread susceptibility that could partly explain the vulnerability of all AI/AN populations to invasive infections caused by encapsulated organisms (*Haemophilus influenzae, Streptococcus pneumoniae*) that is well described but incompletely understood (*49*). A similar mechanism could be involved with GAS disease. The application of more recently developed techniques that measure host immune response to bacterial infections may offer additional insights, which can guide future efforts at GAS vaccine development and control (*50*).

Given the disparity in iGAS among AI/AN, it is reasonable to consider a host genetic predisposition to invasive infections. In a world of rapidly evolving techniques for genetic and epigenetic testing and the emerging potential for gene-targeted therapies, exploring the possibility of a genetic predisposition seems prudent to guide potential therapies for this marginalized population.

## Conclusions

The medical and public health communities need to address the effects of iGAS among the ≈3 million AI/AN in the United States. The first step should address the lack of population-based studies of Indigenous Americans. The Indian Health Service needs to support further investigations into iGAS to fulfill its mission “[t]o raise the physical, mental, social, and spiritual health of American Indians and Alaska Natives to the highest level” (https://www.ihs.gov/aboutihs).

We recommend mandatory reporting of iGAS in regions with substantial numbers of AI/AN. This reporting would encourage smaller healthcare facilities to monitor iGAS outbreaks and improve surveillance in smaller, vulnerable populations. Greater capture of isolates is integral to using population-based epidemiologic studies to better identify risk factors for iGAS specific to AI/AN. We also propose large-scale sequencing and phylogenetic studies of GAS *emm* types to clarify the migration of strains within and among AI/AN and determination of unique host immune responses to GAS among AI/AN to guide control efforts. A tailored approach might be necessary for AI/AN or individual tribes, but better evidence would inform community-based interventions to reduce the incidence of iGAS and lay the foundation for multivalent GAS vaccines to protect communities from the most harmful GAS strains.

Appendix Incidence rates of invasive group A streptococcal disease in indigenous and nonindigenous communities.
