# Supplementary material for: Disparate Effects of Invasive Group A Streptococcus on Native Americans
Source: Emerg Infect Dis. 2020 Sep;26(9):1971–7. doi: 10.3201/eid2609.181169 (PMC7454107; doi:10.3201/eid2609.181169)
Supplement: Appendix — Incidence rates of invasive group A streptococcal disease in indigenous and nonindigenous communities. [file 18-1169-Techapp-s1.pdf]

Article DOI: https://doi.org/10.3201/eid2609.181169

Disparate Effects of Invasive Group A *Streptococcus* on Native Americans

Appendix

Appendix Table. Comparing incidence rates of invasive group A streptococcal disease between indigenous and nonindigenous communities

Reference*	Years	Location	Indigenous community	Indigenous rate†	Nonindigenous rate†
Hoge et al. (*1*)	1985–1990	Southwest, US	American Indians	36.5	2.7
Benjamin et al. (*2*)	1982–1991	New Mexico, US	American Indians	13.3	1.7‡
Rudolph et al. (*3*)	2001–2013	Alaska, US	Alaskan Natives	13.7	3.9
Degani et al. (*4*)	2000–2005	Yukon, Canada	Inuit, Métis, First Nations Canadians	11.0	1.9
Athey et al. (*5*)	2007–2013	Ontario, Canada	First Nations Canadians	9.6 - 18.0	3.6 - 5.0
Bocking et al. (*6*)	2009–2014	Ontario, Canada	First Nations Canadians	56.2	4.0‡
Carapetis et al. (*7*)	1991–1996	N. Territory, Australia	Aboriginal Australians	23.8	4.7
Norton et al. (*8*)	1996–2001	Queensland, Australia	Aboriginal Australians	82.5	10.3
Whitehead et al. (*9*)	2007–2009	Queensland, Australia	Aboriginal Australian children	9.9 - 13.2	2.2 - 3.0
Gear et al. (*10*)	1998–2009	N. Territory, Australia	Aboriginal Australians	40.6	15.8
Boyd et al. (*11*)	2011–2013	N. Territory, Australia	Aboriginal Australians	69.7	8.8
Safar et al. (*12*)	2005–2006	Auckland, New Zealand	Indigenous Maori	21.6	5.3
Steer et al. (*13*)	2004–2005	Fiji	Indigenous Fijians	19.8	13.9
Steer et al.	2005–2007	Fiji	Indigenous Fijians	13.1	2.5

*Applicable references not included in the main text are provided here.
†Rates were crude rates per 100,000. Not all studies provided age-standardized rates or confidence intervals.
‡Rate for nonindigenous population ascertained using data from other acceptable published sources.

References

1. Hoge CW, Schwartz B, Talkington DF, Breiman RF, MacNeill EM, Englender SJ. The changing epidemiology of invasive group A streptococcal infections and the emergence of streptococcal toxic shock-like syndrome. A retrospective population-based study. JAMA. 1993;269:384–9. PubMed https://doi.org/10.1001/jama.1993.03500030082037

2. Benjamin EM, Gershman M, Goldberg BW. Community-acquired invasive group A beta-hemolytic streptococcal infections in Zuni Indians. Arch Intern Med. 1992;152:1881–4. PubMed https://doi.org/10.1001/archinte.1992.00400210103017

3. Rudolph K, Bruce MG, Bruden D, Zulz T, Reasonover A, Hurlburt D, et al. Epidemiology of invasive group A streptococcal disease in Alaska, 2001 to 2013. J Clin Microbiol. 2016;54:134–41. PubMed https://doi.org/10.1128/JCM.02122-15

4. Degani N, Navarro C, Deeks SL, Lovgren M. Invasive bacterial diseases in northern Canada. Emerg Infect Dis. 2008;14:34–40. PubMed https://doi.org/10.3201/eid1401.061522

5. Athey TB, Teatero S, Sieswerda LE, Gubbay JB, Marchand-Austin A, Li A, et al. High incidence of invasive group A streptococcus disease caused by strains of uncommon emm types in Thunder Bay, Ontario, Canada. J Clin Microbiol. 2016;54:83–92. PubMed https://doi.org/10.1128/JCM.02201-15

6. Bocking N, Matsumoto CL, Loewen K, Teatero S, Marchand-Austin A, Gordon J, et al. High incidence of invasive group A streptococcal infections in remote indigenous communities in northwestern Ontario, Canada. Open Forum Infect Dis. 2016;4:ofw243. PubMed https://doi.org/10.1093/ofid/ofw243

7. Carapetis JR, Walker AM, Hibble M, Sriprakash KS, Currie BJ. Clinical and epidemiological features of group A streptococcal bacteraemia in a region with hyperendemic superficial streptococcal infection. Epidemiol Infect. 1999;122:59–65. PubMed https://doi.org/10.1017/S0950268898001952

8. Norton R, Smith HV, Wood N, Siegbrecht E, Ross A, Ketheesan N. Invasive group A streptococcal disease in North Queensland (1996–2001). Indian J Med Res. 2004;119(Suppl):148–51. PubMed

9. Whitehead BD, Smith HV, Nourse C. Invasive group A streptococcal disease in children in Queensland. Epidemiol Infect. 2011;139:623–8. PubMed https://doi.org/10.1017/S0950268810001378

10. Gear RJ, Carter JC, Carapetis JR, Baird R, Davis JS. Changes in the clinical and epidemiological features of group A streptococcal bacteraemia in Australia’s Northern Territory. Trop Med Int Health. 2015;20:40–7.

11. Boyd R, Patel M, Currie BJ, Holt DC, Harris T, Krause V. High burden of invasive group A streptococcal disease in the Northern Territory of Australia. Epidemiol Infect. 2016;144:1018–27. PubMed https://doi.org/10.1017/S0950268815002010

12. Safar A, Lennon D, Stewart J, Trenholme A, Drinkovic D, Peat B, et al. Invasive group A streptococcal infection and vaccine implications, Auckland, New Zealand. Emerg Infect Dis. 2011;17:983–9. PubMed https://doi.org/10.3201/eid/1706.100804

13. Steer AC, Jenney AJ, Oppedisano F, Batzloff MR, Hartas J, Passmore J, et al. High burden of invasive beta-haemolytic streptococcal infections in Fiji. Epidemiol Infect. 2008;136:621–7. PubMed https://doi.org/10.1017/S095026880700917X
